# HeliCis: a DNA motif discovery tool for colocalized motif pairs with periodic spacing

**DOI:** 10.1186/1471-2105-8-418

**Published:** 2007-10-28

**Authors:** Erik Larsson, Per Lindahl, Petter Mostad

**Affiliations:** 1 Wallenberg Laboratory for Cardiovascular Research, Bruna Stråket 16, Sahlgrenska University Hospital, SE-413 45 Göteborg, SWEDEN; 2 Mathematical Sciences, Chalmers University of Technology and Mathematical Sciences, Göteborg University, SE-412 96 Göteborg, SWEDEN

## Abstract

**Background:**

Correct temporal and spatial gene expression during metazoan development relies on combinatorial interactions between different transcription factors. As a consequence, *cis*-regulatory elements often colocalize in clusters termed *cis-*regulatory modules. These may have requirements on organizational features such as spacing, order and *helical phasing *(periodic spacing) between binding sites. Due to the turning of the DNA helix, a small modification of the distance between a pair of sites may sometimes drastically disrupt function, while insertion of a full helical turn of DNA (10–11 bp) between *cis *elements may cause functionality to be restored. Recently, *de novo *motif discovery methods which incorporate organizational properties such as colocalization and order preferences have been developed, but there are no tools which incorporate periodic spacing into the model.

**Results:**

We have developed a web based motif discovery tool, HeliCis, which features a flexible model which allows *de novo *detection of motifs with periodic spacing. Depending on the parameter settings it may also be used for discovering colocalized motifs without periodicity or motifs separated by a fixed gap of known or unknown length. We show on simulated data that it can efficiently capture the synergistic effects of colocalization and periodic spacing to improve detection of weak DNA motifs. It provides a simple to use web interface which interactively visualizes the current settings and thereby makes it easy to understand the parameters and the model structure.

**Conclusion:**

HeliCis provides simple and efficient *de novo *discovery of colocalized DNA motif pairs, with or without periodic spacing. Our evaluations show that it can detect weak periodic patterns which are not easily discovered using a sequential approach, i.e. first finding the binding sites and second analyzing the properties of their pairwise distances.

## Background

DNA sequence motifs recognized by transcription factors are usually short (~10 bp) with low information content, and matching sequence elements therefore occur randomly in large numbers in the genome. The precise specificity required for correct temporal and spatial transcription during metazoan development relies on combinatorial interactions between binding sites in relatively dense clusters [1]. These clusters, termed *cis*-regulatory modules (CRMs), typically contain sites (*cis*-regulatory elements) for several different transcriptional activators and repressors. CRMs may be unstructured, serving as "billboards" that bring DNA binding proteins into proximity [2]. In this case, the balance of activators and repressors, rather than the order or spacing between factors, is the most important property. They may however also be highly structured, the extreme example being the "enhanceosome"-type CRM, with very little flexibility in the arrangement of recognition sites [3]. Others are more flexible, but with requirements on organizational features such as spacing, order and *helical phasing *between binding sites.

Numerous examples demonstrate the importance of the last feature, the *phase*. A small modification of the distance between a pair of sites may sometimes drastically disrupt function and this is usually attributed to the turning of the DNA helix. In many cases, insertion of a full helical turn of DNA (10–11 bp [4]) between *cis *elements will cause functionality to be restored, as this will cause the same face of the binding protein to be exposed to cofactors and nearby DNA binding factors. The phenomenon has been observed in many studies of single genes, e.g. for AP-1 and RD binding sites in the collagenase-3 promoter [5] as well as for the smooth muscle *α*-actin promoter, where introduction of a 20 bp spacer caused significantly higher reporter activity than a 15 bp spacer [6]. Other examples include the HPV18 enhancer [7], lung surfactant protein B [8], TNF-*α *[9] and Igamma1 [10]. In study of four coregulated *Drosophila *developmental enhancers, a conserved shared organization with pairwise periodic distances between neighboring sites was identified [11]. Periodic signals in distances between neighboring motif pairs have also been observed on a genomic scale in *Drosophila *[12] and other eukaryotes [13].

Significant effort has been put into the problem of *de novo *motif discovery of transcription factor binding sites [14]. The task, often described as a local multiple alignment problem, is difficult due to the degenerate nature of transcription factor recognition sequences. Prediction may sometimes be improved by incorporating organizational features such as colocalization and order preferences into the model, and in recent years several such methods have been proposed [15-19]. The idea of incorporating helical phasing into a motif discovery tool has been suggested [12], but to our knowledge no such tool has yet been devised. We propose a motif sampler which can efficiently discover ordered or unordered colocalized motif pairs *de novo *in DNA sequences. In addition, our tool incorporates an optional periodic spacing model, and we show on simulated data that it can detect weak periodic patterns that are not easily discovered using single motif or colocalization methods.

## Implementation

### Algorithm overview

**Figure 1:**
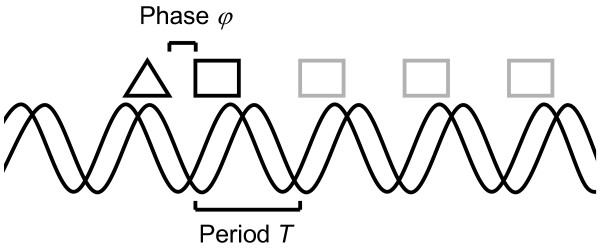
**Schematic drawing of the model structure**. The triangle and rectangle represent the first and second motif respectively. Gray boxes indicate valid locations for the second motif given the position of the first. The "phase" (distance offset) is assumed to be constant over all sequences and is determined by the algorithm.

We propose a *de novo *method for motif discovery, HeliCis, which can find motif pairs separated by a distance (gap) that varies in a periodical manner. More specifically, the distance is modeled as some fixed offset *φ *(the phase) plus a variable integer multiple of the period *T *(Figure 1). Small deviations from exact periodic spacing may optionally be allowed. HeliCis detects patterns which are common to a group of sequences. A typical input would be regulatory DNA from a set of assumedly coregulated genes. The motif pair is assumed to be either present or absent in each sequence and may optionally be allowed to occur on either strand. The period *T *is specified by the user, but the program can be provided with a range of periods to evaluate. Upper and lower boundaries for the distance can be specified. The distance can be allowed to be negative, making it possible to find unordered motif pairs. Our method is not limited to finding periodically distributed binding sites. The flexibility of the algorithm makes the task of finding colocalized motifs (e.g. positioned within 100 bp of one another without periodicity) or motifs with fixed spacing (e.g. always exactly 25 bp from each other) into special cases simply achieved by choosing appropriate parameters. E.g. by setting the period to one, the model will find colocalized motifs without periodicity. Examples of parameter settings for different scenarios are available on the HeliCis home page[20]. The software also incorporates the possibility to take advantage of interspecies conservation by favoring motif placement in highly conserved regions.

### Mathematical model

Let *S *be the set of *N *sequences to be analyzed. Each sequence *s*_*i *_∈ *S*, of length *n*_*i*_, (*i *= 1...*N*) is assumed to contain zero or one motif pair. Below, we refer to motif-containing sequences as being *regulated *and denote this by *R*_*i*_* = true*. The position of the first and second motif in a particular sequence *s*_*i *_is denoted *a*_*i *_and *b*_*i *_respectively. Motifs are modeled as two position frequency matrices *A *and *B*, where *A*_*j*_[*l*] and *B*_*j*_[*l*] denotes the probability of the nucleotide *l *appearing in position *j *of motif A and B respectively. Unregulated sequences are modeled as background sequence, described by an order 0 Markov process with nucleotide frequencies *θ*_0_. Regulated sequences are modeled as a combination of motif and background sequence. The probability of a sequence *s*_*i *_(where *s*_*i*,*j *_denotes the *j*:th base in the sequence) can therefore be written

p(si|Ri=false,θ0)=∏jθ0[si,j]
MathType@MTEF@5@5@+=feaafiart1ev1aaatCvAUfKttLearuWrP9MDH5MBPbIqV92AaeXatLxBI9gBaebbnrfifHhDYfgasaacH8akY=wiFfYdH8Gipec8Eeeu0xXdbba9frFj0=OqFfea0dXdd9vqai=hGuQ8kuc9pgc9s8qqaq=dirpe0xb9q8qiLsFr0=vr0=vr0dc8meaabaqaciaacaGaaeqabaqabeGadaaakeaacqWGWbaCcqGGOaakcqWGZbWCdaWgaaWcbaGaemyAaKgabeaakiabcYha8jabdkfasnaaBaaaleaacqWGPbqAaeqaaOGaeyypa0JaemOzayMaemyyaeMaemiBaWMaem4CamNaemyzauMaeiilaWccciGae8hUde3aaSbaaSqaaiabicdaWaqabaGccqGGPaqkcqGH9aqpdaqeqaqaaiab=H7aXnaaBaaaleaacqaIWaamaeqaaOWaamWaaeaacqWGZbWCdaWgaaWcbaGaemyAaKMaeiilaWIaemOAaOgabeaaaOGaay5waiaaw2faaaWcbaGaemOAaOgabeqdcqGHpis1aaaa@50C8@

and

p(si|Ri=true,A,B,θ0,ai,bi)=QA[ai]⋅QB[bi]⋅∏jθ0[si,j],
MathType@MTEF@5@5@+=feaafiart1ev1aaatCvAUfKttLearuWrP9MDH5MBPbIqV92AaeXatLxBI9gBaebbnrfifHhDYfgasaacH8akY=wiFfYdH8Gipec8Eeeu0xXdbba9frFj0=OqFfea0dXdd9vqai=hGuQ8kuc9pgc9s8qqaq=dirpe0xb9q8qiLsFr0=vr0=vr0dc8meaabaqaciaacaGaaeqabaqabeGadaaakeaacqWGWbaCcqGGOaakcqWGZbWCdaWgaaWcbaGaemyAaKgabeaakiabcYha8jabdkfasnaaBaaaleaacqWGPbqAaeqaaOGaeyypa0JaemiDaqNaemOCaiNaemyDauNaemyzauMaeiilaWIaemyqaeKaeiilaWIaemOqaiKaeiilaWccciGae8hUde3aaSbaaSqaaiabicdaWaqabaGccqGGSaalcqWGHbqydaWgaaWcbaGaemyAaKgabeaakiabcYcaSiabdkgaInaaBaaaleaacqWGPbqAaeqaaOGaeiykaKIaeyypa0Jaemyuae1aaSbaaSqaaiabdgeabbqabaGcdaWadaqaaiabdggaHnaaBaaaleaacqWGPbqAaeqaaaGccaGLBbGaayzxaaGaeyyXICTaemyuae1aaSbaaSqaaiabdkeacbqabaGcdaWadaqaaiabdkgaInaaBaaaleaacqWGPbqAaeqaaaGccaGLBbGaayzxaaGaeyyXIC9aaebeaeaacqWF4oqCdaWgaaWcbaGaeGimaadabeaakmaadmaabaGaem4Cam3aaSbaaSqaaiabdMgaPjabcYcaSiabdQgaQbqabaaakiaawUfacaGLDbaaaSqaaiabdQgaQbqab0Gaey4dIunakiabcYcaSaaa@6EF1@

where

QA[i]=∏k=1WAAk[s1,ii+k−1]θ0[s1,ii+k−1],QB[i]=∏k=1WBBk[s1,ii+k−1]θ0[s1,ii+k−1]
MathType@MTEF@5@5@+=feaafiart1ev1aaatCvAUfKttLearuWrP9MDH5MBPbIqV92AaeXatLxBI9gBaebbnrfifHhDYfgasaacH8akY=wiFfYdH8Gipec8Eeeu0xXdbba9frFj0=OqFfea0dXdd9vqai=hGuQ8kuc9pgc9s8qqaq=dirpe0xb9q8qiLsFr0=vr0=vr0dc8meaabaqaciaacaGaaeqabaqabeGadaaakeaacqWGrbqudaWgaaWcbaGaemyqaeeabeaakmaadmaabaGaemyAaKgacaGLBbGaayzxaaGaeyypa0ZaaebmaeaadaWcaaqaaiabdgeabnaaBaaaleaacqWGRbWAaeqaaOWaamWaaeaacqWGZbWCdaWgaaWcbaGaeGymaeJaeiilaWIaemyAaK2aaSbaaWqaaiabdMgaPbqabaWccqGHRaWkcqWGRbWAcqGHsislcqaIXaqmaeqaaaGccaGLBbGaayzxaaaabaacciGae8hUde3aaSbaaSqaaiabicdaWaqabaGcdaWadaqaaiabdohaZnaaBaaaleaacqaIXaqmcqGGSaalcqWGPbqAdaWgaaadbaGaemyAaKgabeaaliabgUcaRiabdUgaRjabgkHiTiabigdaXaqabaaakiaawUfacaGLDbaaaaaaleaacqWGRbWAcqGH9aqpcqaIXaqmaeaacqWGxbWvdaWgaaadbaGaemyqaeeabeaaa0Gaey4dIunakiabcYcaSiabdgfarnaaBaaaleaacqWGcbGqaeqaaOWaamWaaeaacqWGPbqAaiaawUfacaGLDbaacqGH9aqpdaqeWaqaamaalaaabaGaemOqai0aaSbaaSqaaiabdUgaRbqabaGcdaWadaqaaiabdohaZnaaBaaaleaacqaIXaqmcqGGSaalcqWGPbqAdaWgaaadbaGaemyAaKgabeaaliabgUcaRiabdUgaRjabgkHiTiabigdaXaqabaaakiaawUfacaGLDbaaaeaacqWF4oqCdaWgaaWcbaGaeGimaadabeaakmaadmaabaGaem4Cam3aaSbaaSqaaiabigdaXiabcYcaSiabdMgaPnaaBaaameaacqWGPbqAaeqaaSGaey4kaSIaem4AaSMaeyOeI0IaeGymaedabeaaaOGaay5waiaaw2faaaaaaSqaaiabdUgaRjabg2da9iabigdaXaqaaiabdEfaxnaaBaaameaacqWGcbGqaeqaaaqdcqGHpis1aaaa@8766@

and where *W*_*A *_and *W*_*B *_are widths of the motifs. *a*_*i *_and *b*_*i *_cannot take on arbitrary values but will depend on each other, since we are looking for motif pairs where the distance between the two must follow certain criteria. We use a prior *p*(*a*_*i*_,*b*_*i*_) to reflect this, described below. We also assume there is a fixed prior probability *p(R*_*i *_= *true) *for any sequence to be regulated. For *θ*_0_, *A*_*j*_ and *B*_*j*_ we use Dirichlet priors, with pseudocounts *α*[*l*] proportional to the frequencies of the bases in all the sequences. Our goal is to find values for *R *= (*R*_1_, ..., *R*_*N*_), *a *= (*a*_1_, ..., *a*_*N*_) and *b *= (*b*_1_, ..., *b*_*N*_) which maximize the posterior *p*(*R*,*a*,*b *| *S*). To accomplish this we use an algorithm based on the Gibbs sampling principle for motif discovery [21], which makes use of the predictive update version of the Gibbs sampler [22].

Given a partitioning of the sequences into motifs and background (*a*, *b *and *R*) we can calculate the total observed counts of nucleotide *l *in the background (*c*_0_[*l*]) and in the different positions *i *of motif A (*c*_*A*,*i*_[*l*]) and motif B (*c*_*B*,*i*_[*l*]). Sequences where *R*_*i *_= 0 are assumed to contain only background sequence. We can then estimate *A*, *B*, and *θ*_0 _as the expectation of *p*(*A*, *B*, *θ*_0 _| *R*, *a*, *b*, *S*):

Ai[l]=cA,i[l]+α[l]∑lcA,i[l]+∑lα[l],Bi[l]=cB,i[l]+α[l]∑lcB,i[l]+∑lα[l]
 MathType@MTEF@5@5@+=feaafiart1ev1aaatCvAUfKttLearuWrP9MDH5MBPbIqV92AaeXatLxBI9gBaebbnrfifHhDYfgasaacH8akY=wiFfYdH8Gipec8Eeeu0xXdbba9frFj0=OqFfea0dXdd9vqai=hGuQ8kuc9pgc9s8qqaq=dirpe0xb9q8qiLsFr0=vr0=vr0dc8meaabaqaciaacaGaaeqabaqabeGadaaakeaacqWGbbqqdaWgaaWcbaGaemyAaKgabeaakmaadmaabaGaemiBaWgacaGLBbGaayzxaaGaeyypa0ZaaSaaaeaacqWGJbWydaWgaaWcbaGaemyqaeKaeiilaWIaemyAaKgabeaakmaadmaabaGaemiBaWgacaGLBbGaayzxaaGaey4kaSccciGae8xSde2aamWaaeaacqWGSbaBaiaawUfacaGLDbaaaeaadaaeqaqaaiabdogaJnaaBaaaleaacqWGbbqqcqGGSaalcqWGPbqAaeqaaOWaamWaaeaacqWGSbaBaiaawUfacaGLDbaaaSqaaiabdYgaSbqab0GaeyyeIuoakiabgUcaRmaaqababaGae8xSde2aamWaaeaacqWGSbaBaiaawUfacaGLDbaaaSqaaiabdYgaSbqab0GaeyyeIuoaaaGccqGGSaalcqWGcbGqdaWgaaWcbaGaemyAaKgabeaakmaadmaabaGaemiBaWgacaGLBbGaayzxaaGaeyypa0ZaaSaaaeaacqWGJbWydaWgaaWcbaGaemOqaiKaeiilaWIaemyAaKgabeaakmaadmaabaGaemiBaWgacaGLBbGaayzxaaGaey4kaSIae8xSde2aamWaaeaacqWGSbaBaiaawUfacaGLDbaaaeaadaaeqaqaaiabdogaJnaaBaaaleaacqWGcbGqcqGGSaalcqWGPbqAaeqaaOWaamWaaeaacqWGSbaBaiaawUfacaGLDbaaaSqaaiabdYgaSbqab0GaeyyeIuoakiabgUcaRmaaqababaGae8xSde2aamWaaeaacqWGSbaBaiaawUfacaGLDbaaaSqaaiabdYgaSbqab0GaeyyeIuoaaaaaaa@808A@

θ0[l]=c0[l]+α[l]∑lc0[l]+∑lα[l]
 MathType@MTEF@5@5@+=feaafiart1ev1aaatCvAUfKttLearuWrP9MDH5MBPbIqV92AaeXatLxBI9gBaebbnrfifHhDYfgasaacH8akY=wiFfYdH8Gipec8Eeeu0xXdbba9frFj0=OqFfea0dXdd9vqai=hGuQ8kuc9pgc9s8qqaq=dirpe0xb9q8qiLsFr0=vr0=vr0dc8meaabaqaciaacaGaaeqabaqabeGadaaakeaaiiGacqWF4oqCdaWgaaWcbaGaeGimaadabeaakmaadmaabaGaemiBaWgacaGLBbGaayzxaaGaeyypa0ZaaSaaaeaacqWGJbWydaWgaaWcbaGaeGimaadabeaakmaadmaabaGaemiBaWgacaGLBbGaayzxaaGaey4kaSIae8xSde2aamWaaeaacqWGSbaBaiaawUfacaGLDbaaaeaadaaeqaqaaiabdogaJnaaBaaaleaacqaIWaamaeqaaOWaamWaaeaacqWGSbaBaiaawUfacaGLDbaaaSqaaiabdYgaSbqab0GaeyyeIuoakiabgUcaRmaaqababaGae8xSde2aamWaaeaacqWGSbaBaiaawUfacaGLDbaaaSqaaiabdYgaSbqab0GaeyyeIuoaaaaaaa@51B2@

As in other Gibbs motif samplers, an iterative update/sampling procedure is applied. One of the sequences, *s*_*i*_, is removed from the alignment by setting *R*_*i *_= 0. Given values for *A*, *B*, and *θ*_0 _according to the formulas above, new values for *R*_*i*_, *a*_*i *_and *b*_*i *_are determined by sampling from *p*(*R*_*i*_, *a*_*i*_, *b*_*i *_| *A*, *B*, *θ*_0_, *S*) using the following steps: Bayes formula on odds form gives that

p(Ri=false|si,θ0,A,B)p(Ri=true|si,θ0,A,B)=p(si|Ri=false,θ0)p(si|Ri=true,θ0,A,B)⋅p(Ri=false)p(Ri=true)
 MathType@MTEF@5@5@+=feaafiart1ev1aaatCvAUfKttLearuWrP9MDH5MBPbIqV92AaeXatLxBI9gBaebbnrfifHhDYfgasaacH8akY=wiFfYdH8Gipec8Eeeu0xXdbba9frFj0=OqFfea0dXdd9vqai=hGuQ8kuc9pgc9s8qqaq=dirpe0xb9q8qiLsFr0=vr0=vr0dc8meaabaqaciaacaGaaeqabaqabeGadaaakeaadaWcaaqaaiabdchaWnaabmaabaGaemOuai1aaSbaaSqaaiabdMgaPbqabaGccqGH9aqpcqWGMbGzcqWGHbqycqWGSbaBcqWGZbWCcqWGLbqzcqGG8baFcqWGZbWCdaWgaaWcbaGaemyAaKgabeaakiabcYcaSGGaciab=H7aXnaaBaaaleaacqaIWaamaeqaaOGaeiilaWIaemyqaeKaeiilaWIaemOqaieacaGLOaGaayzkaaaabaGaemiCaa3aaeWaaeaacqWGsbGudaWgaaWcbaGaemyAaKgabeaakiabg2da9iabdsha0jabdkhaYjabdwha1jabdwgaLjabcYha8jabdohaZnaaBaaaleaacqWGPbqAaeqaaOGaeiilaWIae8hUde3aaSbaaSqaaiabicdaWaqabaGccqGGSaalcqWGbbqqcqGGSaalcqWGcbGqaiaawIcacaGLPaaaaaGaeyypa0ZaaSaaaeaacqWGWbaCdaqadaqaaiabdohaZnaaBaaaleaacqWGPbqAaeqaaOGaeiiFaWNaemOuai1aaSbaaSqaaiabdMgaPbqabaGccqGH9aqpcqWGMbGzcqWGHbqycqWGSbaBcqWGZbWCcqWGLbqzcqGGSaalcqWF4oqCdaWgaaWcbaGaeGimaadabeaaaOGaayjkaiaawMcaaaqaaiabdchaWnaabmaabaGaem4Cam3aaSbaaSqaaiabdMgaPbqabaGccqGG8baFcqWGsbGudaWgaaWcbaGaemyAaKgabeaakiabg2da9iabdsha0jabdkhaYjabdwha1jabdwgaLjabcYcaSiab=H7aXnaaBaaaleaacqaIWaamaeqaaOGaeiilaWIaemyqaeKaeiilaWIaemOqaieacaGLOaGaayzkaaaaaiabgwSixpaalaaabaGaemiCaa3aaeWaaeaacqWGsbGudaWgaaWcbaGaemyAaKgabeaakiabg2da9iabdAgaMjabdggaHjabdYgaSjabdohaZjabdwgaLbGaayjkaiaawMcaaaqaaiabdchaWnaabmaabaGaemOuai1aaSbaaSqaaiabdMgaPbqabaGccqGH9aqpcqWG0baDcqWGYbGCcqWG1bqDcqWGLbqzaiaawIcacaGLPaaaaaaaaa@AA0D@

from which we get that

p(Ri=true|si,θi,A,B)=[1+p(si|Ri=false,θ0)p(si|Ri=true,A,B,θ0)⋅1−p(Ri=true)p(Ri=true)]−1,
 MathType@MTEF@5@5@+=feaafiart1ev1aaatCvAUfKttLearuWrP9MDH5MBPbIqV92AaeXatLxBI9gBaebbnrfifHhDYfgasaacH8akY=wiFfYdH8Gipec8Eeeu0xXdbba9frFj0=OqFfea0dXdd9vqai=hGuQ8kuc9pgc9s8qqaq=dirpe0xb9q8qiLsFr0=vr0=vr0dc8meaabaqaciaacaGaaeqabaqabeGadaaakeaacqWGWbaCdaqadaqaaiabdkfasnaaBaaaleaacqWGPbqAaeqaaOGaeyypa0JaemiDaqNaemOCaiNaemyDauNaemyzauMaeiiFaWNaem4Cam3aaSbaaSqaaiabdMgaPbqabaGccqGGSaaliiGacqWF4oqCdaWgaaWcbaGaemyAaKgabeaakiabcYcaSiabdgeabjabcYcaSiabdkeacbGaayjkaiaawMcaaiabg2da9maadmaabaGaeGymaeJaey4kaSYaaSaaaeaacqWGWbaCdaqadaqaaiabdohaZnaaBaaaleaacqWGPbqAaeqaaOGaeiiFaWNaemOuai1aaSbaaSqaaiabdMgaPbqabaGccqGH9aqpcqWGMbGzcqWGHbqycqWGSbaBcqWGZbWCcqWGLbqzcqGGSaalcqWF4oqCdaWgaaWcbaGaeGimaadabeaaaOGaayjkaiaawMcaaaqaaiabdchaWnaabmaabaGaem4Cam3aaSbaaSqaaiabdMgaPbqabaGccqGG8baFcqWGsbGudaWgaaWcbaGaemyAaKgabeaakiabg2da9iabdsha0jabdkhaYjabdwha1jabdwgaLjabcYcaSiabdgeabjabcYcaSiabdkeacjabcYcaSiab=H7aXnaaBaaaleaacqaIWaamaeqaaaGccaGLOaGaayzkaaaaaiabgwSixpaalaaabaGaeGymaeJaeyOeI0IaemiCaa3aaeWaaeaacqWGsbGudaWgaaWcbaGaemyAaKgabeaakiabg2da9iabdsha0jabdkhaYjabdwha1jabdwgaLbGaayjkaiaawMcaaaqaaiabdchaWnaabmaabaGaemOuai1aaSbaaSqaaiabdMgaPbqabaGccqGH9aqpcqWG0baDcqWGYbGCcqWG1bqDcqWGLbqzaiaawIcacaGLPaaaaaaacaGLBbGaayzxaaWaaWbaaSqabeaacqGHsislcqaIXaqmaaGccqGGSaalaaa@985A@

which is used to sample whether *R*_*i *_= *true*. Note that, using (1) and (2), we have

p(si|Ri=true,A,B,θ0)p(si|Ri=false,θ0)=∑ai,bi=1nip(ai,bi)⋅QA[ai]⋅QB[bi].
 MathType@MTEF@5@5@+=feaafiart1ev1aaatCvAUfKttLearuWrP9MDH5MBPbIqV92AaeXatLxBI9gBaebbnrfifHhDYfgasaacH8akY=wiFfYdH8Gipec8Eeeu0xXdbba9frFj0=OqFfea0dXdd9vqai=hGuQ8kuc9pgc9s8qqaq=dirpe0xb9q8qiLsFr0=vr0=vr0dc8meaabaqaciaacaGaaeqabaqabeGadaaakeaadaWcaaqaaiabdchaWnaabmaabaGaem4Cam3aaSbaaSqaaiabdMgaPbqabaGccqGG8baFcqWGsbGudaWgaaWcbaGaemyAaKgabeaakiabg2da9iabdsha0jabdkhaYjabdwha1jabdwgaLjabcYcaSiabdgeabjabcYcaSiabdkeacjabcYcaSGGaciab=H7aXnaaBaaaleaacqaIWaamaeqaaaGccaGLOaGaayzkaaaabaGaemiCaa3aaeWaaeaacqWGZbWCdaWgaaWcbaGaemyAaKgabeaakiabcYha8jabdkfasnaaBaaaleaacqWGPbqAaeqaaOGaeyypa0JaemOzayMaemyyaeMaemiBaWMaem4CamNaemyzauMaeiilaWIae8hUde3aaSbaaSqaaiabicdaWaqabaaakiaawIcacaGLPaaaaaGaeyypa0ZaaabCaeaacqWGWbaCdaqadaqaaiabdggaHnaaBaaaleaacqWGPbqAaeqaaOGaeiilaWIaemOyai2aaSbaaSqaaiabdMgaPbqabaaakiaawIcacaGLPaaaaSqaaiabdggaHnaaBaaameaacqWGPbqAaeqaaSGaeiilaWIaemOyai2aaSbaaWqaaiabdMgaPbqabaWccqGH9aqpcqaIXaqmaeaacqWGUbGBdaWgaaadbaGaemyAaKgabeaaa0GaeyyeIuoakiabgwSixlabdgfarnaaBaaaleaacqWGbbqqaeqaaOWaamWaaeaacqWGHbqydaWgaaWcbaGaemyAaKgabeaaaOGaay5waiaaw2faaiabgwSixlabdgfarnaaBaaaleaacqWGcbGqaeqaaOWaamWaaeaacqWGIbGydaWgaaWcbaGaemyAaKgabeaaaOGaay5waiaaw2faaiabc6caUaaa@872C@

We define the prior *p*(*a*_*i*_,*b*_*i*_) to be proportional to an indicator function *e*(*a*_*i*_,*b*_*i*_) which is zero unless *a*_*i *_and *b*_*i *_represent a pair of motif positions compatible with the assumptions that the motifs are both within the sequence, do not overlap, and have a distance conforming to the assumed periodicity and the assumed possible variation around this periodicity. As described above, the allowed distance is modeled as a fixed phase *φ *plus a variable integer multiple of the period *T *(Figure 1). Specifically, given *W*_*A*_, *W*_*B*_, the period *T*, the phase *φ*, the allowed deviation from exact periodic distance ("noise"), the length of sequence *i *and the minimum and maximum distances, we can for all *i *= 1..*n*_*i *_find all *j *such that *e*(*i*,*j*) = 1, and the value of (8) can be calculated as

∑i=1ni[QA[ai]⋅∑j:e(ai,j)=1QB[j]]∑i=1ni∑j=1nie(i,j).
 MathType@MTEF@5@5@+=feaafiart1ev1aaatCvAUfKttLearuWrP9MDH5MBPbIqV92AaeXatLxBI9gBaebbnrfifHhDYfgasaacH8akY=wiFfYdH8Gipec8Eeeu0xXdbba9frFj0=OqFfea0dXdd9vqai=hGuQ8kuc9pgc9s8qqaq=dirpe0xb9q8qiLsFr0=vr0=vr0dc8meaabaqaciaacaGaaeqabaqabeGadaaakeaadaWccaqaamaaqahabaWaamWaaeaacqWGrbqudaWgaaWcbaGaemyqaeeabeaakmaadmaabaGaemyyae2aaSbaaSqaaiabdMgaPbqabaaakiaawUfacaGLDbaacqGHflY1daaeqbqaaiabdgfarnaaBaaaleaacqWGcbGqaeqaaOWaamWaaeaacqWGQbGAaiaawUfacaGLDbaaaSqaaiabdQgaQjabcQda6iabdwgaLjabcIcaOiabdggaHnaaBaaameaacqWGPbqAaeqaaSGaeiilaWIaemOAaOMaeiykaKIaeyypa0JaeGymaedabeqdcqGHris5aaGccaGLBbGaayzxaaaaleaacqWGPbqAcqGH9aqpcqaIXaqmaeaacqWGUbGBdaWgaaadbaGaemyAaKgabeaaa0GaeyyeIuoaaOqaamaaqahabaWaaabCaeaacqWGLbqzcqGGOaakcqWGPbqAcqGGSaalcqWGQbGAcqGGPaqkaSqaaiabdQgaQjabg2da9iabigdaXaqaaiabd6gaUnaaBaaameaacqWGPbqAaeqaaaqdcqGHris5aaWcbaGaemyAaKMaeyypa0JaeGymaedabaGaemOBa42aaSbaaWqaaiabdMgaPbqabaaaniabggHiLdaaaOGaeiOla4caaa@6DA4@

Secondly, we get that *p*(*a*_*i *_| *R*_*i *_= *true*, *A*, *B*, *θ*_0_, S) is proportional to

QA[ai]⋅∑k∈e(i,ai)QB[k],
 MathType@MTEF@5@5@+=feaafiart1ev1aaatCvAUfKttLearuWrP9MDH5MBPbIqV92AaeXatLxBI9gBaebbnrfifHhDYfgasaacH8akY=wiFfYdH8Gipec8Eeeu0xXdbba9frFj0=OqFfea0dXdd9vqai=hGuQ8kuc9pgc9s8qqaq=dirpe0xb9q8qiLsFr0=vr0=vr0dc8meaabaqaciaacaGaaeqabaqabeGadaaakeaacqWGrbqudaWgaaWcbaGaemyqaeeabeaakmaadmaabaGaemyyae2aaSbaaSqaaiabdMgaPbqabaaakiaawUfacaGLDbaacqGHflY1daaeqbqaaiabdgfarnaaBaaaleaacqWGcbGqaeqaaOWaamWaaeaacqWGRbWAaiaawUfacaGLDbaaaSqaaiabdUgaRjabgIGiolabdwgaLjabcIcaOiabdMgaPjabcYcaSiabdggaHnaaBaaameaacqWGPbqAaeqaaSGaeiykaKcabeqdcqGHris5aOGaeiilaWcaaa@49FD@

so if *R*_*i *_= *true*, a value for *a*_*i *_can be sampled by using probabilities proportional to the numbers (10). Finally, *b*_*i *_can be sampled by noting that given *R*_*i *_= *true *and a value for *a*_*i*_, the probabilities for valid values of *b*_*i *_according to *e*(*i*, *a*_*i*_) are proportional to *Q*_*B*_[*b*_*i*_].

The algorithm is initiated by setting all *R*_*i *_= *false*. The update/sampling procedure described above is then performed for each sequence *s*_*i*_, *i *= 1...*N*. When all *R*_*i*_, *a*_*i *_and *b*_*i *_have been updated, the alignment is scored according to

F=log⁡p(S|A,B,θ0,a,b,R)p(S|θ0,R=(false,...,false))=∑k=1WA∑lcA,k[l]log⁡(Ak[l]θ0(l))+∑k=1WB∑lcB,k[l]log⁡(Bk[l]θ0(l))
 MathType@MTEF@5@5@+=feaafiart1ev1aaatCvAUfKttLearuWrP9MDH5MBPbIqV92AaeXatLxBI9gBaebbnrfifHhDYfgasaacH8akY=wiFfYdH8Gipec8Eeeu0xXdbba9frFj0=OqFfea0dXdd9vqai=hGuQ8kuc9pgc9s8qqaq=dirpe0xb9q8qiLsFr0=vr0=vr0dc8meaabaqaciaacaGaaeqabaqabeGadaaakqaabeqaaiabdAeagjabg2da9iGbcYgaSjabc+gaVjabcEgaNnaalaaabaGaemiCaa3aaeWaaeaacqWGtbWucqGG8baFcqWGbbqqcqGGSaalcqWGcbGqcqGGSaaliiGacqWF4oqCdaWgaaWcbaGaeGimaadabeaakiabcYcaSiabdggaHjabcYcaSiabdkgaIjabcYcaSiabdkfasbGaayjkaiaawMcaaaqaaiabdchaWnaabmaabaGaem4uamLaeiiFaWNae8hUde3aaSbaaSqaaiabicdaWaqabaGccqGGSaalcqWGsbGucqGH9aqpcqGGOaakcqWGMbGzcqWGHbqycqWGSbaBcqWGZbWCcqWGLbqzcqGGSaalcqGGUaGlcqGGUaGlcqGGUaGlcqGGSaalcqWGMbGzcqWGHbqycqWGSbaBcqWGZbWCcqWGLbqzcqGGPaqkaiaawIcacaGLPaaaaaaabaGaeyypa0ZaaabmaeaadaaeqaqaaiabdogaJnaaBaaaleaacqWGbbqqcqGGSaalcqWGRbWAaeqaaOWaamWaaeaacqWGSbaBaiaawUfacaGLDbaaaSqaaiabdYgaSbqab0GaeyyeIuoakiGbcYgaSjabc+gaVjabcEgaNjabcIcaOmaalaaabaGaemyqae0aaSbaaSqaaiabdUgaRbqabaGcdaWadaqaaiabdYgaSbGaay5waiaaw2faaaqaaiab=H7aXnaaBaaaleaacqaIWaamaeqaaOGaeiikaGIaemiBaWMaeiykaKcaaiabcMcaPaWcbaGaem4AaSMaeyypa0JaeGymaedabaGaem4vaC1aaSbaaWqaaiabdgeabbqabaaaniabggHiLdGccqGHRaWkdaaeWaqaamaaqababaGaem4yam2aaSbaaSqaaiabdkeacjabcYcaSiabdUgaRbqabaGcdaWadaqaaiabdYgaSbGaay5waiaaw2faaaWcbaGaemiBaWgabeqdcqGHris5aOGagiiBaWMaei4Ba8Maei4zaCMaeiikaGYaaSaaaeaacqWGcbGqdaWgaaWcbaGaem4AaSgabeaakmaadmaabaGaemiBaWgacaGLBbGaayzxaaaabaGae8hUde3aaSbaaSqaaiabicdaWaqabaGccqGGOaakcqWGSbaBcqGGPaqkaaGaeiykaKcaleaacqWGRbWAcqGH9aqpcqaIXaqmaeaacqWGxbWvdaWgaaadbaGaemOqaieabeaaa0GaeyyeIuoaaaaa@B0C2@

We are interested in finding values which maximize *p*(*R*, *a*, *b *| *S*), which approximately corresponds to maximizing *F *above. Having completed a full iteration of the update/sampling procedure, sampling continues at the first sequence. The algorithm stops when the same *F *has been observed several times in a row or when the maximum number of iterations is reached. To avoid getting stuck in local maxima, the algorithm is restarted several times. It is also systematically restarted with different settings of the phase *φ *(all values between 0...*T*-1 are evaluated), as this parameter is not updated during each run of the algorithm and therefore has to be determined exhaustively.

To avoid that the algorithm finds "shifted versions" of the actual motifs, a type of shift jump is introduced. Each time the score *F *is improved, possible shifts of the motifs are found, defined by adding or subtracting some integer to all *a*_*i *_and *b*_*i*_. For each of the possible shifts (a*, b*), we calculate *F*. If a better score is encountered, the positions are updated and used as a starting point for the next update/sampling iteration.

For simplicity, we have described the case where motif pairs are assumed to occur only on the forward strand. Our method optionally permits both forward and reverse strands to be searched. In this case, the sampling distribution and the calculation of the posterior probability for *R *is extended to included both strands. Optionally, information about conservation between species can be used to favor placement of motifs in evolutionarily conserved regions. In this case, instead of single sequences, pairwise alignments of orthologous sequences are loaded into the program. Gaps are removed from the "base" sequences to ensure that correct distances are maintained. The fraction of conserved bases over windows the same size as the motifs is calculated for each possible motif position. The sampling distributions are then weighted according to this vector. A similar strategy is implemented in [23]. The same vector is also used to exclude regions from being searched. This allows the sampler to be restarted after convergence to search for a new set of non-overlapping binding sites.

### Implementation and user interface

**Figure 2:**
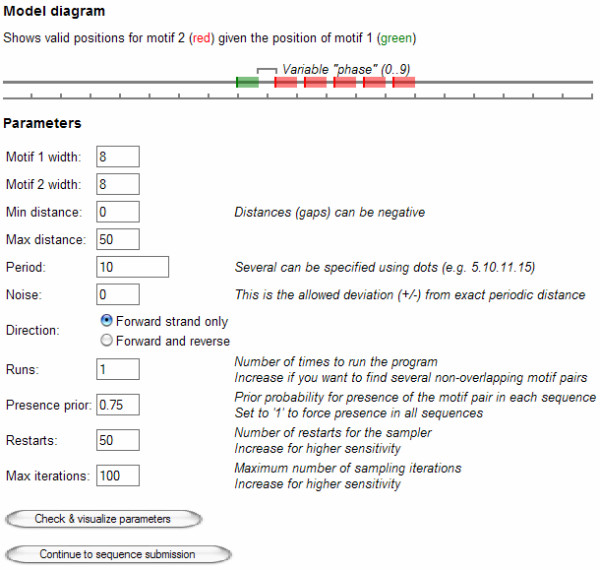
**Web interface screenshot, showing the parameter setup screen**. The schematic shows valid positions for motif 2 given the position of motif one. The image is dynamically generated to reflect the current parameter settings.

The main algorithm is implemented in Matlab while time critical functions are written in the C language. These can be downloaded for local use (see Additional File 1). HeliCis is also available through a web interface[20] which provides several templates to simplify parameter setup. To make it easier to understand the function of the different parameters, these are visualized using an interactive schematic figure which is updated to reflect the current settings (Figure 2). The web interface is implemented in php and the source files can be made available upon request.

## Results

### Performance vs. motif information content

The performance was evaluated on synthetic sequence datasets. Ordered pairs of SRF (CArG) and ETS binding sites, generated from raw TRANSFAC [24] weight matrices (M01007 and M00771), were planted into sets of 15 random sequences of length 400 bp. The choice of matrices was arbitrary, although these factors have been shown to cooperatively regulate certain genes [25]. One motif pair was assigned to each sequence and the distance between each pair was set to a uniformly random multiple (n = 0...4) of the helical period (10 bp) plus a 5 bp offset. The binding sites were thus both colocalized and periodically spaced. The TRANSFAC CArG matrix is based on 54 occurrences and the central 12 bases were used when generating the test sequences (the core CArG motif is 10 bp long). The ETS matrix is 12 bp long and based on 48 occurrences. Raw counts were converted into relative frequencies and bases were randomly selected according to this distribution. Several sequence sets with increasingly weaker motifs were generated by varying the number of pseudocounts between 0 and 4. The information content of the resulting matrices was calculated. Evaluation sequence sets are available both as supplementary information (see Additional File 2) and for download on the HeliCis homepage [20].

**Figure 3:**
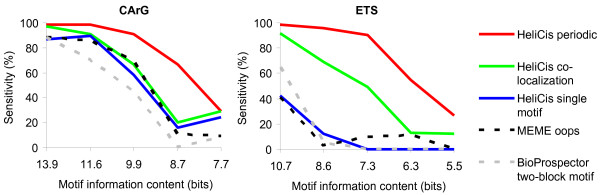
**Performance on synthetic sequence datasets containing colocalized and periodically spaced CArG and ETS motifs with varying information content**. HeliCis with different settings was compared to MEME and BioProspector. The information content of the motifs was gradually reduced by varying the number of pseudocounts and the sensitivity of the different tools was determined by calculating the fraction of correctly identified motifs. Results are from 5 averaged trials.

HeliCis with default settings for periodic spacing (period 10, motif distance 0...50 bp), HeliCis with colocalization settings (period 1, distance 0...50 bp) and HeliCis with single motif settings were compared to an established single motif discovery tool based on the EM algorithm, MEME [26], and a motif discovery tool based on Gibbs sampling, BioProspector [27]. The latter was run in "two-block" mode, searching for motif pairs with a maximum gap of 50 bp. All were configured to search the forward strand only with a fixed motif width of 12 bp, and with forced presence of a motif in each sequence (oops = "one occurrence per sequence" model in MEME, "-a 1" switch for BioProspector, "-p 1" switch for HeliCis). The quality of the resulting alignments was determined by calculating the fraction of correctly identified sites (Figure 3). Results shown are average values from five independent trials where the sequence sets were regenerated each time. It should be noted that BioProspector, unlike HeliCis and MEME, cannot be forced to detect exactly one occurrence per sequence, but will often assign several motifs per sequence. This should be taken into account when evaluating the results, as this model may be slightly disadvantageous on this dataset.

The CArG motif has high information content and all tested tools performed reasonably well on this motif before pseudocounts were added. However, the sensitivity of HeliCis with periodic and colocalization settings was still higher, reaching 99 % and 97 % respectively, as opposed to 88 % for MEME and BioProspector. As the information content of the motifs was lowered, the ability of the periodic model to make use of the periodicity in the data became obvious and the other methods were outperformed. When the already weak ETS motif was obscured by added pseudocounts, HeliCis in colocalization mode quickly lost its ability to make use of this motif to improve detection of the CArG box.

The ETS motif was not efficiently detected using any of the single motif methods, and this is where the advantages of the HeliCis model were most obvious. BioProspector in two-block mode was able to draw some advantage of the proximity to the stronger CArG motif and reached 65 % sensivity with no added pseudocounts, to be compared with ~42 % for MEME and HeliCis in single motif mode. The corresponding result for HeliCis in colocalization mode was 92 %, and the advantage was even bigger when the information content of the motifs was reduced. On the ETS motif, HeliCis in periodic mode had considerably higher sensitivity than all the other tested methods throughout the series.

### Performance vs. fraction of sequences containing motifs

**Figure 4:**
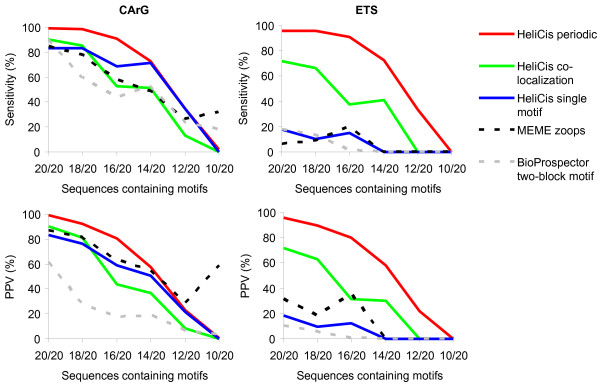
**Performance on synthetic sequence datasets with varying motif coverage**. Datasets of 20 sequences with colocalized and periodically spaced CArG and ETS motifs were generated. The proportion of sequences containing the motifs was gradually reduced, thus making them increasingly difficult to detect. HeliCis with different settings was compared to MEME and BioProspector. The plots show sensitivity and positive predictive value (PPV = TP/(TP + FP)). Results are from 5 averaged trials.

In a second evaluation, sets of 20 sequences containing artificially planted CArG and ETS motifs were generated as described above. However, this time the information content of the motif matrices was kept constant (one pseudocount added). Instead, the fraction of sequences containing motifs was gradually reduced from 20/20 to 10/20, thus making them increasingly difficult to detect. In this case, the tools were not forced to detect motifs in all sequences (zoops = "zero or one occurrences per sequence" model in MEME, default for BioProspector and HeliCis). Other settings were as described above. To account for false positive predictions, a PPV score (positive predictive value, i.e. the fraction of predicted sites which are correct) was calculated, in addition to sensitivity. The results, shown in Figure 4, are average values from 5 independent trials.

Again, the less informative ETS motif benefited considerably from the HeliCis model, both with periodic and colocalization settings. This motif was only sporadically detected by MEME, BioProspector and HeliCis with single motif settings, while HeliCis in periodic mode reached 91 % sensitivity when 16/20 sequences contained the motifs. When the fraction of motif-containing sequences was high (20/20 to 16/20) also the CArG motif was detected with higher sensitivity by HeliCis in periodic mode compared to the other tested tools.

In the most challenging dataset, with motifs in 10 out of 20 sequences, HeliCis was not able to detect any motifs. However, both MEME and BioProspector could sporadically detect the CArG motif with average sensitivity scores of 32 % and 18 % respectively. MEME generally performed well in the PPV plots, reflecting that it was less prone to assigning false positive motifs in non-motif containing sequences. BioProspector does not have the possibility to limit the number of detected two-block motifs to maximum one per sequence. Due to a larger number of false positive predictions it therefore scored unfavorably in the PPV plots. It should be noted that its two-block model was occasionally able to detect the difficult ETS motif with high sensitivity, however, the average performance was still similar to the single motif methods.

## Discussion

We have described a novel tool for *de novo *discovery of regulatory DNA motifs, HeliCis, available for local use and through a web interface[20]. Our method can efficiently detect motif pairs which are spatially colocalized in regulatory DNA. It is based on a flexible probabilistic model which optionally allows *de novo *discovery of motif pairs with periodic spacing (helical phasing). A large number of experimental studies show the importance of helical phasing in regulatory regions. The ability to detect such patterns *de novo *without prior knowledge of recognition sequences may be useful in the study of coregulated CRMs.

Our results show that HeliCis is able to efficiently take advantage of the synergistic effects of colocalization to improve sensitivity to weak DNA patterns. HeliCis in colocalization mode was evaluated on planted ETS and CArG motifs which were colocalized with a spacer of random variable length. The weaker ETS motif was detected with far better accuracy compared to other tested methods, and this can be attributed to the ability of our method to make use of the nearby stronger CArG motif to improve sensitivity. Detection of the CArG motif also benefited from the ETS-motif, although to a lesser extent. Sensitivity was further improved in a drastic way by running HeliCis in periodic mode. Both the CArG and the ETS motif benefited considerably from this reduction of the search space. Importantly, this shows that the method is capable of finding weak periodic patterns which are not readily detected using a "sequential" approach, i.e. first detecting single motifs and second analyzing their spacing properties.

One limitation of our model is that the motifs widths are fixed. Some Gibbs sampling algorithms handle this using an alternative scoring function and restarts using several widths [21] or the "fragmentation algorithm [28]," while others use a fixed width [15,27]. TF binding sites are usually within the 8–12 bp range and we have found results to be quite robust to changes in this parameter as long as the motif width is not set too short. Results were nearly identical when HeliCis was applied to the test sets in this paper using a 10 bp motif width instead of the default 12 bp (data not shown).

HeliCis models the intermotif distance as a variable integer multiple of the period *T *plus a fixed "phase" (offset) *φ *= 0...*T*-1. The phase is determined exhaustively by restarting the sampler several times, leading execution time to be proportional to the chosen period. A desirable improvement would be to determine the phase during execution of the algorithm rather than to use restarts. If several periods other than the default 10 bp are to be evaluated, more restarts are required and the algorithm can become computationally demanding. However, the current implementation normally does not cause problems with sequence sets of reasonable size. With 15 400 bp sequences, execution time with the periodic model (10 bp period) is typically around 10 minutes on a low-end processor (Pentium 4 2.4 GHz). The execution time in each iteration theoretically scales linearly with the number of sequences, the total amount of sequence data, the motif length and the maximum motif distance. In practice, as long as each individual sequence is not to long (<1000 bp), the number of sequences is the most important factor (data not shown). Some parameters in the web interface have been slightly limited to avoid overloading the server, but no such limitations are present in the downloadable version.

## Conclusion

HeliCis is a flexible and efficient tool for *de novo *discovery of colocalized DNA motif pairs. It incorporates structural features such as ordered or unordered colocalization and periodic spacing. Our evaluations show that it can detect weak periodic patterns which cannot be easily discovered by others means. It is available both for local use and through a simple web interface.

## Availability and requirements

Project name: HeliCis

Project home page: 

Operating system: Platform independent

Programming language: Matlab, C

License: Free for academic and non-profit researchers. Contact the authors for commercial licensing.

## Authors' contributions

The functional specification of the method was prepared by EL and PL. The mathematical model and algorithm was designed and implemented by EL. The web interface was implemented by EL. The manuscript was drafted by EL and PM with contributions from PL. All authors read and approved the final manuscript.

## Supplementary Material

Additional file 1 Matlab and C source files. This archive contains source files and instructions for compilation. Click here for file

Additional file 2 Evaluation sequences. This archive contains evaluation sequence datasets and the Matlab scripts used for generating them. Click here for file

## References

[B1] Davidson EH (2006). The Regulatory Genome: Gene Regulatory Networks In Development and Evolution.

[B2] Kulkarni MM, Arnosti DN (2003). Information display by transcriptional enhancers. Development.

[B3] Carey M (1998). The enhanceosome and transcriptional synergy. Cell.

[B4] Wang JC (1979). Helical repeat of DNA in solution. Proc Natl Acad Sci U S A.

[B5] D'Alonzo RC, Selvamurugan N, Karsenty G, Partridge NC (2002). Physical interaction of the activator protein-1 factors c-Fos and c-Jun with Cbfa1 for collagenase-3 promoter activation. J Biol Chem.

[B6] Mack CP, Thompson MM, Lawrenz-Smith S, Owens GK (2000). Smooth muscle alpha-actin CArG elements coordinate formation of a smooth muscle cell-selective, serum response factor-containing activation complex. Circ Res.

[B7] Bouallaga I, Massicard S, Yaniv M, Thierry F (2000). An enhanceosome containing the Jun B/Fra-2 heterodimer and the HMG-I(Y) architectural protein controls HPV 18 transcription. EMBO Rep.

[B8] Alam MN, Berhane K, Boggaram V (2002). Lung surfactant protein B promoter function is dependent on the helical phasing, orientation and combinatorial actions of cis-DNA elements. Gene.

[B9] Barthel R, Tsytsykova AV, Barczak AK, Tsai EY, Dascher CC, Brenner MB, Goldfeld AE (2003). Regulation of tumor necrosis factor alpha gene expression by mycobacteria involves the assembly of a unique enhanceosome dependent on the coactivator proteins CBP/p300. Mol Cell Biol.

[B10] Dryer RL, Covey LR (2005). A novel NF-kappa B-regulated site within the human I gamma 1 promoter requires p300 for optimal transcriptional activity. J Immunol.

[B11] Erives A, Levine M (2004). Coordinate enhancers share common organizational features in the Drosophila genome. Proc Natl Acad Sci U S A.

[B12] Makeev VJ, Lifanov AP, Nazina AG, Papatsenko DA (2003). Distance preferences in the arrangement of binding motifs and hierarchical levels in organization of transcription regulatory information. Nucleic Acids Res.

[B13] Ioshikhes I, Trifonov EN, Zhang MQ (1999). Periodical distribution of transcription factor sites in promoter regions and connection with chromatin structure. Proc Natl Acad Sci U S A.

[B14] Tompa M, Li N, Bailey TL, Church GM, De Moor B, Eskin E, Favorov AV, Frith MC, Fu Y, Kent WJ, Makeev VJ, Mironov AA, Noble WS, Pavesi G, Pesole G, Regnier M, Simonis N, Sinha S, Thijs G, van Helden J, Vandenbogaert M, Weng Z, Workman C, Ye C, Zhu Z (2005). Assessing computational tools for the discovery of transcription factor binding sites. Nat Biotechnol.

[B15] Gupta M, Liu JS (2005). De novo cis-regulatory module elicitation for eukaryotic genomes. Proc Natl Acad Sci U S A.

[B16] Marsan L, Sagot MF (2000). Algorithms for extracting structured motifs using a suffix tree with an application to promoter and regulatory site consensus identification. J Comput Biol.

[B17] Segal E, Sharan R (2005). A discriminative model for identifying spatial cis-regulatory modules. J Comput Biol.

[B18] Zhou Q, Wong WH (2004). CisModule: de novo discovery of cis-regulatory modules by hierarchical mixture modeling. Proc Natl Acad Sci U S A.

[B19] Thompson W, Palumbo MJ, Wasserman WW, Liu JS, Lawrence CE (2004). Decoding human regulatory circuits. Genome Res.

[B20] HeliCis website. http://lymphomics.wall.gu.se/helicis.

[B21] Lawrence CE, Altschul SF, Boguski MS, Liu JS, Neuwald AF, Wootton JC (1993). Detecting subtle sequence signals: a Gibbs sampling strategy for multiple alignment. Science.

[B22] Liu J (1992). The collapsed Gibbs sampler and other issues: with applications to a protein binding problem. Research Report No R-426, Dept Statistics, Harvard Univ.

[B23] Thompson W, Rouchka EC, Lawrence CE (2003). Gibbs Recursive Sampler: finding transcription factor binding sites. Nucleic Acids Res.

[B24] Wingender E (1994). Recognition of regulatory regions in genomic sequences. J Biotechnol.

[B25] Wang Z, Wang DZ, Hockemeyer D, McAnally J, Nordheim A, Olson EN (2004). Myocardin and ternary complex factors compete for SRF to control smooth muscle gene expression. Nature.

[B26] Bailey TL, Elkan C (1994). Fitting a mixture model by expectation maximization to discover motifs in biopolymers. Proc Int Conf Intell Syst Mol Biol.

[B27] Liu X, Brutlag DL, Liu JS (2001). BioProspector: discovering conserved DNA motifs in upstream regulatory regions of co-expressed genes. Pac Symp Biocomput.

[B28] Neuwald AF, Liu JS, Lawrence CE (1995). Gibbs motif sampling: detection of bacterial outer membrane protein repeats. Protein Sci.

